# HSV-1 infection suppresses *TGF-β1* and *SMAD3* expression in human corneal epithelial cells

**Published:** 2008-09-03

**Authors:** Yuhong Nie, Dongmei Cui, Zhujuan Pan, Jiangyun Deng, Qiang Huang, Kaili Wu

**Affiliations:** Zhongshan Ophthalmic Center, State Key Laboratory of Ophthalmology, Sun Yat-sen University, Guangzhou, P.R. China

## Abstract

**Purpose:**

The present study was undertaken to investigate whether transforming growth factor-β (*TGF-β*) isoforms (TGF-β1, TGF-β2, and TGF-β3) and SMADs (*SMAD2* and *SMAD3*) are involved in herpes simplex virus type 1 (HSV-1) corneal infection.

**Methods:**

Human corneal epithelial cells (HCE) were infected with HSV-1 at a multiplicity of infection of 5. Cell morphological changes were observed under phase-contrast microscopy. Levels of mRNA for *TGF-β* isoforms 1, 2, and 3 as well as for *SMAD2* and *SMAD3* were measured by reverse transcription polymerase chain reaction (RT–PCR) at 0 h, 4 h, 8 h, 12 h, and 24 h after infection. Protein expression of TGF-β1, TGF-β2, SMAD3, and phospho-SMAD3 were analyzed by indirect immunofluorescence at 0 h, 12 h, and 24 h post-infection (p.i.) in HCE cells. Protein expression of TGF-β1 was also evaluated by ELISA.

**Results:**

Following HSV-1 infection, a cytopathic effect in HCE cells was observed at 8 h p.i. and became significant at 24 h p.i. Compared with normal cells, the mRNA levels of *TGF-β1* in HSV-1 infected HCE cells decreased significantly at 8 h, 12 h, and 24 h p.i. (p<0.01), and the expression of *SMAD3* was also dramatically decreased 12 h and 24 h p.i. (p<0.01). No noticeable changes were found as a result of infection with respect to levels of *TGF-β2*, *TGF-β3*, and *SMAD2* in HCE cells. Protein expression of TGF-β1, SMAD3, and phospho-SMAD3 decreased in infected cells at 12 h and 24 h p.i. compared with normal cells, but TGF-β2 had no change.

**Conclusions:**

*TGF-β1* and *SMAD3* may be involved in the pathology of corneal diseases associated with HSV-1 infection.

## Introduction

Herpes simplex virus type 1 (HSV-1) is a large, enveloped, double-stranded DNA virus with a genome of approximately 150 kbp. HSV-1 is widespread in the human population and commonly causes infections of the skin or mucosal surfaces. Occasionally, it can cause serious diseases such as sporadic encephalitis and ocular infections [1,2]. In the eye, HSV-1 infection usually results in blepharitis, conjunctivitis, corneal epithelial keratitis, and ulcerative and/or stromal keratitis [3]. The pathologies of these diseases are associated with several events such as the infiltration of neutrophils and mononuclear lymphocytes and the expression of growth factors, proinflammatory factors, and cytokines, which include transforming growth factor-β (TGF-β), IL-2, IL-6, IL-8, TNF-α, and interferon-β (IFN-β) [4-6]. These studies suggest that growth factors and cytokines are extremely important in regulating the body’s reaction to viral infection.

TGF-β is a superfamily of cytokines, which affect a range of biological processes such as cell growth, differentiation, matrix production, migration, and apoptosis [7]. Furthermore, the TGF-β pathway is an important target for several viral proteins that interfere with signal transduction and transcription control in infected cells [8-11]. Upon activation of the TGF-β signaling pathway, TGF-β family members bind to the TGF-β type II receptor (TβR-II). TβR-II then recruits and phosphorylates TGF-β type I receptors (TβR-I), which in turn phosphorylates the intracellular effectors (i.e., SMAD2 and SMAD3). Subsequently, SMAD2 and SMAD3 complexes, which are associated with SMAD4, are translocated into the nucleus and regulate the transcription of target genes [7,12,13]. A previous study demonstrated that TGF-β isoforms are expressed in the human cornea [14,15], and TGF-β is believed to be one of the major factors involved in cell migration in the cornea and corneal wound healing [16-18]. Furthermore, TGF-β signaling through the SMAD pathway is activated in response to corneal wounds in which the basement membrane is removed [16]. Earlier studies suggested that TGF-β might be important in the pathology of various disease processes involved with viral infection. This has been demonstrated for a variety of viruses including cytomegalovirus (CMV), human immunodeficiency virus (HIV), canine distemper virus, rhinovirus, HSV-1, and human T-cell leukemia virus (HTLV) [8,9,19-22]. Corneal epithelial cells are the first line of defense against microbial infection and against further damage to the underlying stroma. Therefore, we must understand the role of TGF-β in the pathology of viral infection in the corneal epithelium. It is reasonable to suppose that TGF-β and SMADs play a critical role in the pathology of HSV-1 infection in the cornea. The present study was undertaken to examine whether TGF-β isoforms and SMADs (SMAD2 and SMAD3) are involved in HSV-1 corneal epithelial infection in vitro.

## Methods

### Cell culture

The human corneal epithelial cell line (HCEC) that we used was described previously [23]. Cells were cultured in DMEM/high glucose supplemented with 10% fetal bovine serum (FBS; Hyclone, Logan, UT), 10 ng/ml human epidermal growth factor (EGF; Sigma, St Louis, MO), 5 μg/ml insulin, 5 μg/ml human transferrin (Sigma), and 0.4 μg/ml hydrocortisone (Gibco BRL, Grand Island, NY). The cells were incubated at 37 °C in a 5% CO_2_-95% air incubator. Experiments were performed when cells were at 80%-90% confluence.

### Virus infection

Stocks of the HSV-1 (F strain) used in this study were propagated on HEp-2 cells grown in DMEM/F12, which contained 10% newborn bovine serum. The titer of virus stocks was determined according to a previously described method [24]. After cells were grown to 80%-90% confluence, cells were infected at a multiplicity of infection (MOI) of 5. After 1 h of adsorption at 37 °C with intermittent rocking, the inoculum was removed, and the medium was replaced with serum-free DMEM/high glucose. At the indicated times, cells were harvested for further experiments. To confirm virus infection, two virus genes (i.e., DNA polymerase and *VP16*) of HSV-1 were examined by reverse transcription polymerase chain reaction (RT–PCR) using the primers listed in Table 1. Two genes were detected in HSV-1 infected cells, which implied that HCE cells were successfully infected by HSV-1.

**Table 1:** Primer sequences and length of amplicons.

**Gene**	**Primer sequences**	**Product size (bp)**
*TGF-β1*	forward: 5′-GGGACTATCCACCTGCAAGA-3′	239
	reverse: 5′-CCTCCTTGGCGTAGTAGTCG-3′	
*TGF-β2*	forward: 5′-GTGGAGGTGCCATCAATA-3′	499
	reverse: 5′-GAGGAGCGACGAAGAGTA-3′	
*TGF-β3*	forward: 5′-CAA AGGGCTCTGGTGGTC-3′	216
	reverse: 5′-CGGGTGCTGTTGTAAAGTG-3′	
*SMAD3*	forward: 5′-AGGAGAAATGGTGCGAGA A-3′	197
	reverse: 5′-CCACAGGCGGCAGTAGAT-3′	
*SMAD2*	forward: 5′-TCACAGTCATCATGAACTCAAGG-3′	471
	reverse: 5′-TGTGACGCATGGAAGGTCTCTC-3′	
*DNA polymerase*	forward: 5′-ATCAACTTCGACTGGCCCTT-3′	179
	reverse: 5′-CCGTACATGTCGATGTTCAC-3′	
*VP16*	forward: 5′-GGTCGCAACAGAGGCAGTCA-3′	418
	reverse: 5′-CCCGAACGCACCCAAATC-3′	
*GAPDH*	forward: 5′-GCACCGTCAAGGCTGAGAAC-3′	138
	reverse: 5′- TGGTGAAGACGCCAGTGGA-3′	

### RNA isolation and reverse transcription polymerase chain reaction analysis

Cells were harvested and washed with phosphate buffered saline (PBS). Total RNA was isolated with TRIzol reagent (Invitrogen, Carlsbad, CA) according to the manufacturer’s instructions. The quantity and quality of total RNA were estimated by spectrophotometry and agarose electrophoresis. Subsequently, RNA was reverse-transcribed into cDNA using a RevertAid^TM^ First Strand cDNA synthesis kit (Fermentas, Glen Burnie, MD). cDNA was then amplified by GoTaq® Green Master mix (Promega, Madison, WI) using the specific primers listed in Table 1. The PCR products were electrophoresed in GoldView^TM^ stained 2% agarose gels (SBS Genetech, Beijing, China). Quantification of the bands was performed using a BioImaging System (UVP, Upland, CA) and Gel-pro software (Media Cybernetics, Bethesda, MD), and the level of mRNA was expressed as the ratio of integrated optical density (IOD) of specific PCR products over *GAPDH* IOD.

### Indirect immunofluorescence

HCE cells were cultured on a glass coverslip in 12 well chamber dishes and infected with HSV-1 as described above. At the indicated times, changes in cellular morphology were photographed using a phase-contrast microscope. Slide-mounted cells were used for indirect immunofluorescence analysis according to the method described previously [25]. The cells were blocked by endogenous peroxidase-blocking solution and followed by goat serum (each for 10 min at 37 °C). After blocking nonspecific binding, cells were incubated with rabbit anti-human monoclonal/polyclonal antibodies that recognize TGF-β1 (Santa Cruz, Delaware Avenue, CA), TGF-β2 (Santa Cruz), SMAD3, and phospho-SMAD3 (both from Cell Signaling, Danvers, MA) at 4 °C overnight. Cells were then incubated with FITC-conjugated secondary goat anti-rabbit IgG (Zhongshan Goldenbridge, Beijing, China) at 37 °C for 1 h. Prior to mounting, cells were stained with propidium iodide (PI) for 10 min. Cells were then observed using a confocal laser scanning microscope (Carl Zeiss, Jena, Germany). Cells incubated with PBS (instead of the first antibody) were used as negative controls.

### Measurement of TGF-β1 protein in human corneal epithelial cells by ELISA

At 0 h, 12 h, and 24 h p.i., HSV-1 infected HCE cells were lysed with lysate buffer (Pierce, Rockford, IL). The samples were sonicated and centrifuged at 12,000 rpm for 30 min at 4 °C to remove cellular debris. Protein content in the supernatant was determined by the bicinchoninic acid method using BSA as the standard. The TGF-β1 levels of cell homogenate were assayed using a specific TGF-β1 enzyme-liked immunosorbent assay kit (Boster, Wuhan, China), and human TGF-β1 was used to construct a standard curve. The amount of TGF-β1 protein in the cell was normalized to the total amount of cellular protein. Absorbance values were read at 450 nm by an ELISA enzyme-labeled device.

### Statistical analysis

Statistical analysis of data was performed by one-way ANOVA and a Student–Newman–Keuls test to determine statistically significant differences (p<0.05) between uninfected and HSV-1 infected cells.

## Results

### Morphological changes of HSV-1 infected human corneal epithelial cells

**Figure 1:**
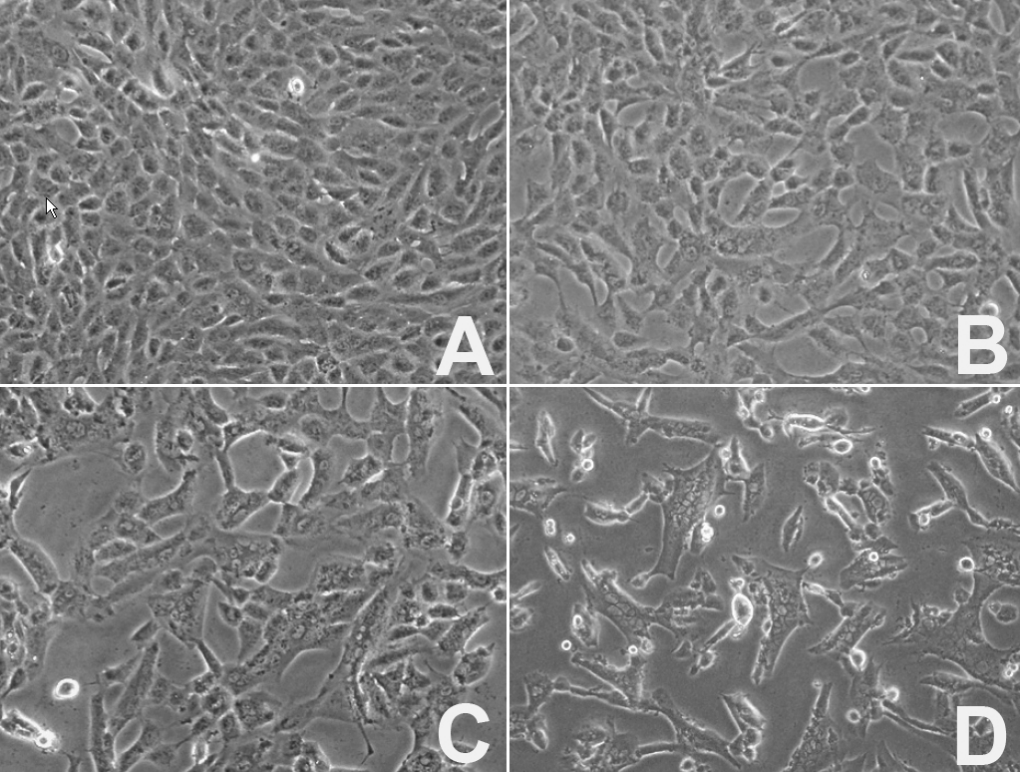
Morphological changes of human corneal epithelial cells infected with HSV-1. **A**: Normal human corneal epithelial cells exhibited a cobblestone appearance. **B**: The cytopathic effect could be seen at 8 h p.i. The space between infected cells increased. After cells were infected with HSV-1 for 12 h (**C**) and 24 h (**D**), the cobblestone appearance disappeared and many giant multinucleated cells could be seen. Magnification: 200X.

Cell morphological changes were observed under phase-contrast microscopy. Normal HCE cells exhibited a typical cobblestone appearance (Figure 1A). Following HSV-1 infection and up to 8 h p.i., the cell morphology of infected groups was similar to the uninfected group. Compared with control cells, a cytopathic effect (CPE) in HCE cells could be observed at 8 h and 12 h p.i. (Figure 1B,C). The space between infected cells increased, and the cobblestone appearance disappeared. At 24 h p.i., most of the infected cells exhibited a CPE (dead cells were observed floating in the medium), and many giant multinucleated cells could be seen (Figure 1D).

### Expression of TGF-β isoforms in HSV-1 infected human corneal epithelial cells in vitro

**Figure 2:**
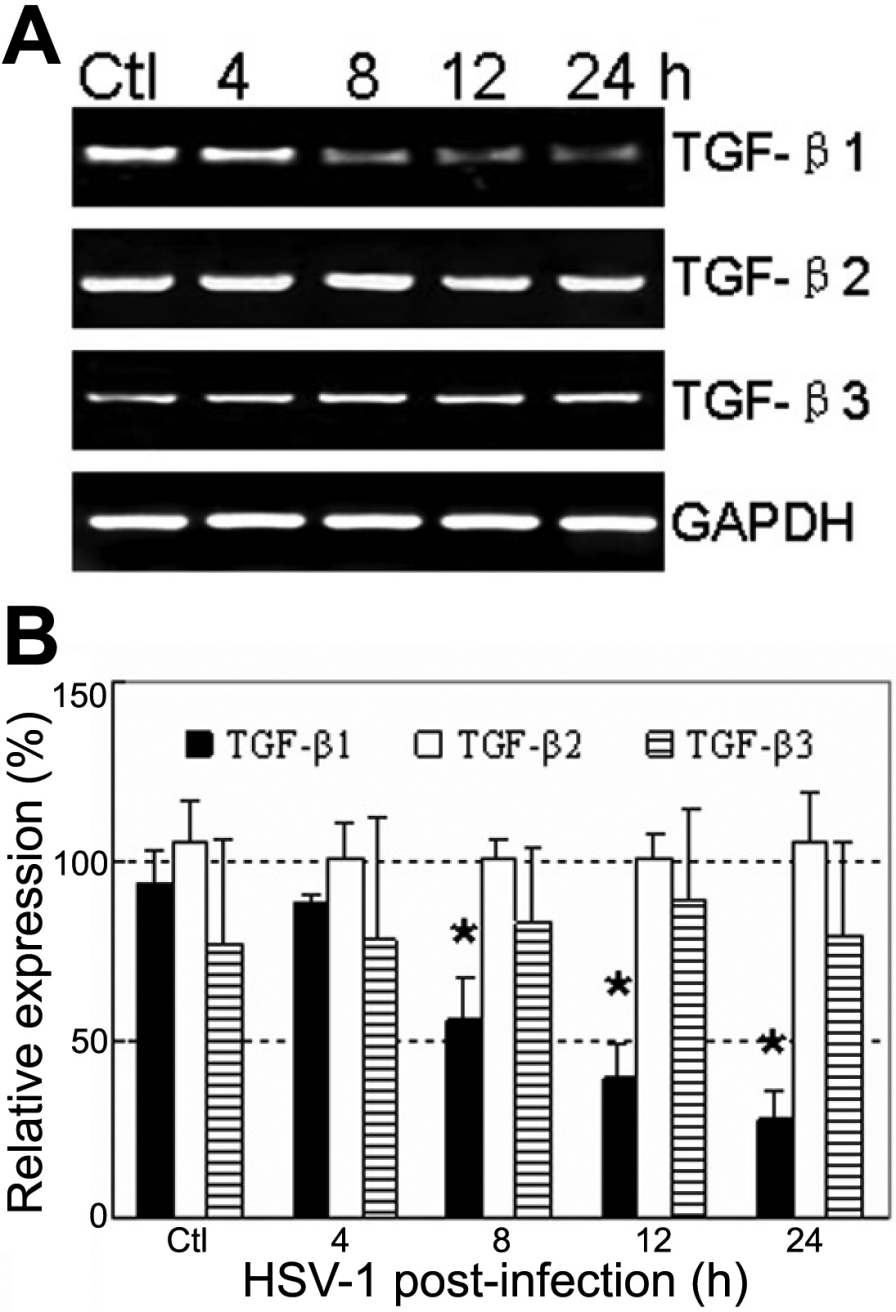
Reverse transcription polymerase chain reaction analysis of the *TGF-β* isoforms in human corneal epithelial cells infected with HSV-1. **A**: Products of RT-PCR that were run on 2% agarose gel electrophoresis. The intensities of *TGF-β1* bands decreased significantly at 8 h, 12 h, and 24 h p.i., while that of *TGF-β2* and *TGF-β3* bands unchanged. *GAPDH* was used as an internal control. **B**: The level of mRNA was expressed as the ratio of integrated optical density (IOD) of specific PCR products over *GAPDH* IOD. Each data was the mean value of three independent experiments. Single asterisks indicate significant differences (p<0.05).

First, the mRNA level of TGF-β isoforms (i.e., *TGF-β1*, *TGF-β2*, and *TGF-β3*) in HCE cells infected with HSV-1 was estimated using RT–PCR (Figure 2). The mRNA level of *TGF-β1* decreased significantly by 40.3%, 57.3%, and 70.4% at 8 h, 12 h, and 24 h p.i., respectively, when compared with uninfected cells (p<0.01). However, mRNA profiles of *TGF-β2* and *TGF-β3* in infected cells at 8 h, 12 h, and 24 h p.i were similar to that of uninfected cells (p>0.05).

**Figure 3:**
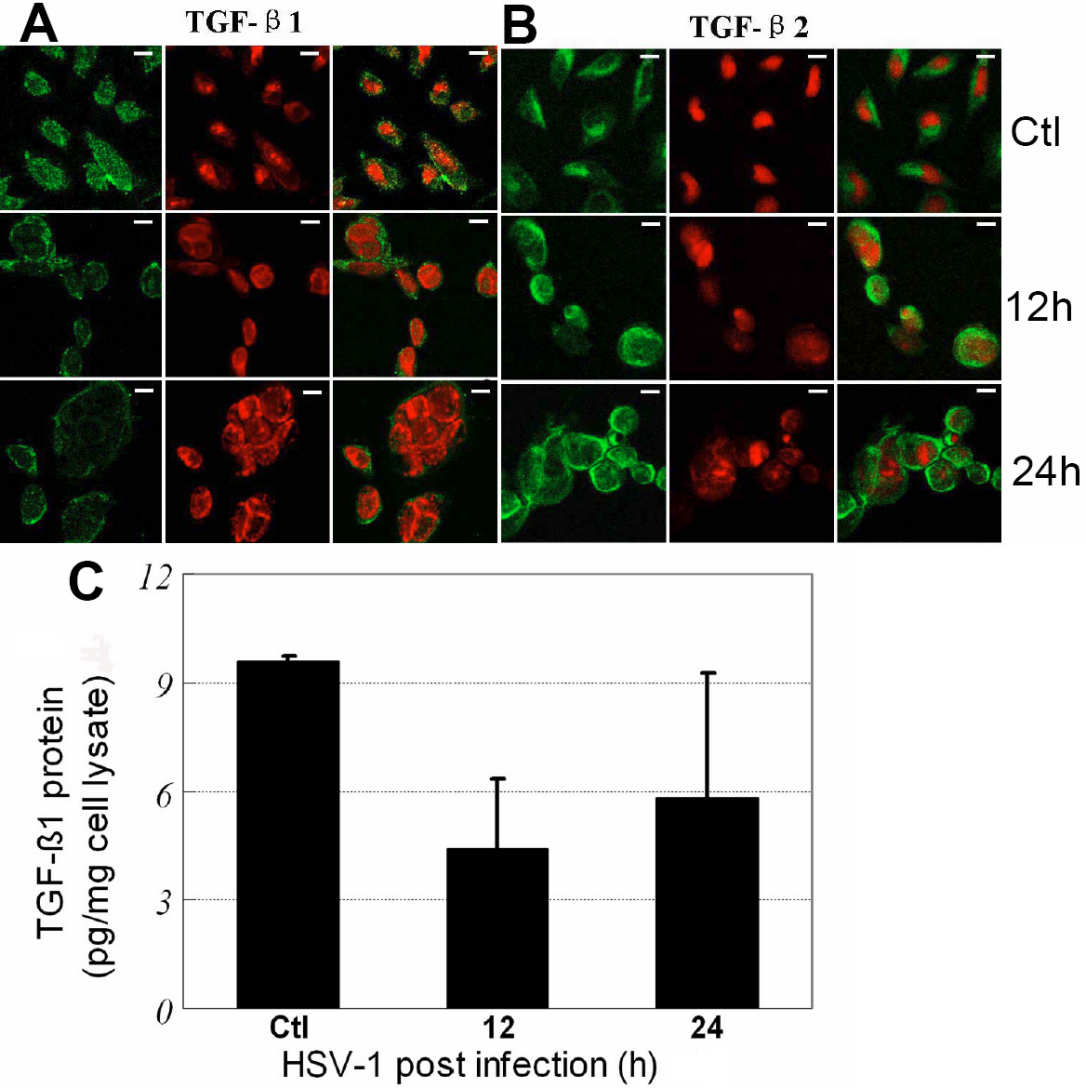
Protein expression of TGF-β1 and TGF-β2 in human corneal  epithelial cells infected with HSV-1. In **A** and **B**, indirect  immunofluorescence analysis was used to find the protein expression in  cells. FITC marked the secondary antibody (green; left), and PI dyed the nucleus (red; middle). Merged images were showed at the right of **A** and **B**. Scale bar: 10 μm. **C**: The expression of TGF-β1 by ELISA in HCE cells infected with HSV-1 was measured at 0 h, 12 h, and 24 h p.i. Significant decreases of the TGF-β1 protein in cell lysates were seen in both 12 h and 24 h post-infected cells (p<0.05). Each data was the mean value of four independent assays.

To further verify the results of PCR, indirect immunofluorescence was used to observe the changes of TGF-β1and TGF-β2 protein expression in HCE cells infected with HSV-1 (Figure 3). The intensity of immunostaining for TGF-β1 decreased at 12 h and 24 h p.i. compared with the control (Figure 3A). The decrease of TGF-β1 protein in HSV-1 infected HCE cells was also found by ELISA measurement (Figure 3C). Significant decreases in the levels of TGF-β1 protein were observed using two immunomethods. However, compared with normal cells, TGF-β2 protein remained present in infected cells at both 12 h and 24 h p.i. when we examined the cells by immunocytochemical staining (Figure 3B).

### Expression of *SMAD2* and *SMAD3* in HSV-1 infected HCE cells

**Figure 4:**
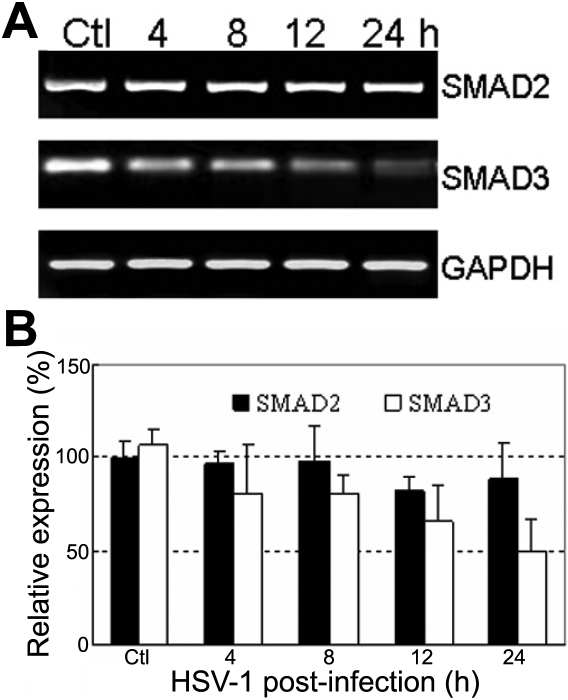
Reverse transcription polymerase chain reaction analysis of *SMAD2* and *SMAD3* in human corneal epithelial cells infected with HSV-1. **A**: Agarose gel pattern of RT–PCR products. The band intensities of *SMAD3*, not *SMAD2*, decreased during the period of post-infection. *GAPDH* was used as an internal control. **B**: The level of mRNA was expressed as the ratio of IOD of specific PCR products over the *GAPDH* gene IOD. The mean values of three independent experiments are shown. Single asterisks indicate significant differences (p<0.05).

The expression of *SMAD2* and *SMAD3* in HCE cells infected with HSV-1 was detected by RT–PCR (Figure 4). This study found a clear reduction in mRNA level coding for *SMAD3* in HSV-1 infected cells. Compared with normal cells, *SMAD3* mRNA levels decreased significantly by 37.5% (12 h p.i.) and 53.1% (24 h p.i.; p<0.01) in infected cells. However, the mRNA levels of *SMAD2* remained unchanged during the course of infection (p>0.05).

**Figure 5:**
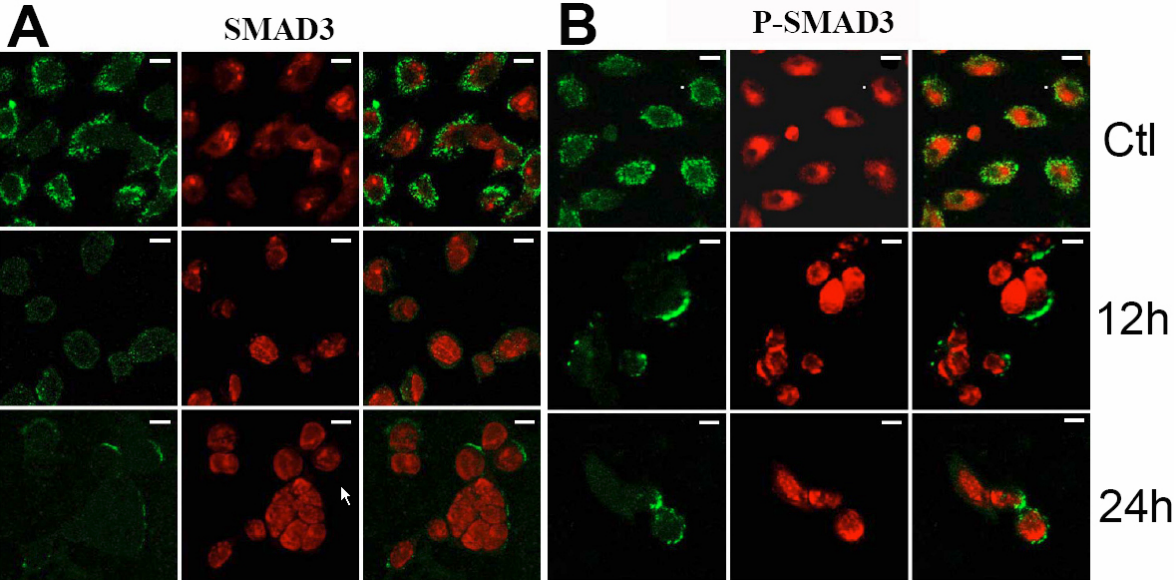
Colocalization of SMAD3 and phospho-SMAD3 protein in human corneal epithelial cells. FITC marked the secondary antibody (green; left), and PI dyed the nucleus (red; middle). Merged images were showed at the right of **A** and **B**. Both SMAD3 (**A**) and phospho-SMAD3 (**B**) were more weakly expressed at 12 h and 24 h p.i. compared to the uninfected cells. Scale bar: 10 μm.

To examine whether the down-regulation of *SMAD3* mRNA also results in a reduction in protein level, SMAD3 and phospho-SMAD3 protein expressions during HSV-1 infection were analyzed by immunocytochemistry. Compared with normal cells, protein expression of SMAD3 and phospho-SMAD3 in infected cells was weaker at 12 h and 24 h p.i. (Figure 5).

## Discussion

The cornea contains three principal cell types, epithelial cells, keratocytes, and endothelial cells. Previous studies have shown that corneal epithelial cells are capable of supporting efficient HSV-1 replication [26,27]. Balliet et al. [28] reported that a recombinant HSV-1 virus, KOS-CMVGFP, expressing enhanced green fluorescent protein (EGFP) could infect mice as efficiently as a wild-type virus. They found that fluorescence was observed in eyes as distinct small foci on the cornea at day 1 p.i., and that the fluorescence spread throughout the eye between days 1 and 3 p.i. Finally, the foci grew larger and coalesced, resulting in large, dendritic corneal lesions. Consistent with the studies described above, our work also demonstrated that the HCE cell is highly permissive to HSV-1 infection in vitro. When HCE cells were infected with HSV-1 at a MOI of 5, a cytopathic effect was observed at 8 h p.i. HSV-1 infection caused an increase in the number of dead cells, which may be the reason for the dendritic keratitis observed in vivo. Furthermore, we also observed expression of two viral genes (DNA polymerase and *VP16*) in infected cells by RT–PCR, which implies that HSV-1 caused a productive infection of HCE cells. Therefore, HCE cells are susceptible to HSV-1 infection, and it can provide a useful in vitro model for research of HSV-1 infection in the cornea.

TGF-β isoforms and SMAD family members have been identified in mammalian cells. In the eye, TGF-β isoforms are expressed in different ocular tissues [14,15,29]. In the cornea, SMAD2 and SMAD4 were expressed and translocated into the nuclei, and SMAD7 was overexpressed during corneal epithelial wound repair [16,30]. In the cultured retinal pigment epithelial cell line (D407), TGF-β can stimulate the translocation of SMAD2 (but not SMAD1) from the cytoplasm into the nuclei [31]. Therefore, TGF-β isoforms and SMADs may play important roles in the pathogenesis of ocular diseases. However, there is limited research on the effect of TGF-β isoforms and SMADs in cells infected by HSV-1. Accordingly, the objective of the present study was to investigate whether the expression of TGF-β isoforms and SMADs in HCE cells is affected by HSV-1 infection in vitro.

The effect of viral infection on TGF-β expression has been described for several viruses including HIV, CMV, and HSV-1 in other tissues [19,32,33]. In CMV infection, TGF-β1 was detected in increasing amounts in infected human foreskin fibroblast and astrocyte supernatants, and TGF-β1 transcription was significantly increased when compared to that of uninfected cells [22,33]. In vitro HSV-1 infection of human mononuclear cells resulted in a significant time-dependent increase in the release of TGF-β1 protein into supernatants [19]. These studies showed that virus infection could induce the overexpression of TGF-β1 with respect to protein expression and mRNA levels. However, in a study on mouse cornea infected with HSV-1, Hu et al. [4] showed that levels of *TGF-β* mRNA decreased in inflamed corneas. Our study demonstrated that the expression of *TGF-β1* at both the mRNA and protein level was down-regulated in HCE cells infected by HSV-1 at 8 h p.i. and beyond. However, during the course of HSV-1 infection, the transcription of *TGF-β2* and *TGF-β3* remained unchanged compared to uninfected cells. These results suggested that *TGF-β* expression in response to HSV-1 infection is specific to cell type.

The current study also showed that HSV-1 infection caused a decline in the transcription of *SMAD3* in HCE cells but had no effect on the expression of *SMAD2*. Similarly, by confocal laser scanning microscopy, HSV-1 infected HCE cells displayed weak immunostaining for SMAD3 and phospho-SMAD3. Although measuring protein levels with a quantitative method such as western blot would provide more convincing evidence of protein expression change, the immunostaining result was consistent with the data of RT–PCR analysis for *SMAD3*. These results suggested that *SMAD3* decreased in both mRNA and protein levels in HSV-1 infected HCE cells.

It has been demonstrated that in virus infections, *TGF-β* could be regulated by the SMAD subfamily. In HPV infected cells, viral E7 oncoprotein blocks through its constitutive interactions with SMAD2, SMAD3, and SMAD4, both SMAD transcriptional activity and the ability of TGF-β to inhibit DNA synthesis [10]. E6 oncoprotein of HPV-5 inhibits SMAD3 transactivation by interacting with SMAD3, destabilizing the SMAD3/SMAD4 complex, and inducing the degradation of both proteins [34]. Virus proteins also interfere with TGF-β signaling via SMAD proteins as observed in HTLV-1 infected ATL cells [8] and in Kaposi's sarcoma-associated herpes virus infection [11]. These results show that suppression of SMAD-mediated TGF-β signaling in virus infected cells might contribute to the carcinogenesis. The present study focuses on HSV-1 infected corneal epithelial cells, which characterizes recurrent inflammation of the cornea in vivo. The fundamental physiologic roles of SMAD3 are involved in the processes of tissue repair and fibrosis [35]. Decreased *SMAD3* expression could reduce formation and nuclear import of transcriptionally active SMAD heterocomplexes and decrease transcription of TGF-β1 regulated target genes, which result in reduced inflammatory cell infiltrates, reduced auto-induction of *TGF-β*, and reduced elaboration of collagen [36]. This may be the cause of the observed decreases of TGF-β1 and SMAD3 in HSV-1 infected HCE cells in this study, which occurred as an in vivo inflammatory process.

The interplay between HSV-1 and its host involves numerous factors, and the virus employs several mechanisms to combat many antiviral responses enacted by the infected cell [37]. Expression of *TGF-β1* and *SMAD3* in HSV-1 infected HCE cells decreased in this study, which suggested that they may be involved in corneal diseases that are associated with HSV-1 infection. The specific function of TGF-β1 and SMAD3 in HSV-1 corneal infection requires further investigation.
